# ^99m^Tc-Radiolabeled TPGS Nanomicelles Outperform ^99m^Tc-Sestamibi as Breast Cancer Imaging Agent

**DOI:** 10.1155/2019/4087895

**Published:** 2019-04-23

**Authors:** Fiorella C. Tesan, Melisa B. Nicoud, Mariel Nuñez, Vanina A. Medina, Diego A. Chiappetta, María J. Salgueiro

**Affiliations:** ^1^Universidad de Buenos Aires, Facultad de Farmacia y Bioquímica, Cátedra de Física, CABA, Argentina; ^2^Laboratorio de Biología Tumoral e Inflamación, Instituto de Investigaciones Biomédicas (BIOMED), Consejo Nacional de Investigaciones Científicas y Técnicas (CONICET), Universidad Católica Argentina (UCA), Instituto de Investigaciones Biomédicas (BIOMED), CABA, Argentina; ^3^Consejo Nacional de Investigaciones Científicas y Técnicas, Argentina; ^4^Universidad de Buenos Aires, Facultad de Farmacia y Bioquímica, Cátedra de Tecnología Farmacéutica I, CABA, Argentina

## Abstract

D-*α*-Tocopheryl polyethylene glycol 1000 succinate (TPGS) is a Food and Drug Administration (FDA) approved biomaterial that can form nanosized micelles in aqueous solution. TPGS micelles stand as an interesting system to perform drug delivery as they can carry lipophilic drugs and overcome P glycoprotein efflux as well. Therefore, TPGS micelles combined with other copolymers have been reported in many cancer research studies as a carrier for therapeutic drugs. Their ability to reach tumoral tissue can also be exploited to develop imaging agents with diagnostic application. A radiolabeling method with ^99m^Tc for TPGS nanosized micelles and their biodistribution in a healthy animal model as well as their pharmacokinetics and radiolabeling stability *in vivo* was previously reported. The aim of this work was to evaluate the performance of this radioactive probe as a diagnostic imaging agent compared to routinely available SPECT radiopharmaceutical, ^99m^Tc-sestamibi. A small field of view gamma camera was used for scintigraphy studies using radiolabeled TPGS micelles in two animal models of breast cancer: syngeneic 4T1 murine cell line (injected in BALB/c mice) and chemically NMU-induced (Sprague-Dawley rats). *Ex vivo* radioactivity accumulation in organs of interest was measured by a solid scintillation counter, and a semiquantitative analysis was performed over acquired images as well. Results showed an absence of tumoral visualization in 4T1 model for both radioactive probes by gamma camera imaging. On the contrary, NMU-induced tumors had a clear tumor visualization by scintigraphy. A higher tumor/background ratio and more homogeneous uptake were found for radiolabeled TPGS micelles compared to ^99m^Tc-sestamibi. In conclusion, ^99m^Tc-radiolabeled TPGS micelles might be a potential SPECT imaging probe for diagnostic purposes.

## 1. Introduction

Imaging techniques have become essential diagnostic tools in clinical practice. Interestingly, small animal imaging has grown over the last years and positioned as a crucial part of biomedical preclinical research. It constitutes a noninvasive modality that provides qualitative and quantitative information, allowing to explore physiological processes spatially and temporarily [1]. This approach becomes even more relevant towards imaging probe and even new drug development, since preclinical results based on imaging detection should be more readily translatable to clinical area [1, 2]. Additionally, preclinical imaging agrees with the three R's concept in which replacement, reduction, and refinement are the requested principles for laboratory animal use in research [3].

On the other hand, nanomedicine research has grown exponentially over the past decades and its applications are of great interest for human health. Unique characteristics of nanomaterials make them suitable to be exploited in specific areas such as therapy and diagnosis of cancer diseases. Moreover, enhanced permeability and retention effect (EPR, passive targeting) and molecularly driven active targeting are the bedrock in which nanosized systems rely on to address tumoral tissue [4, 5]. For instance, D-*α*-tocopheryl polyethylene glycol 1000 succinate (TPGS) is a Food and Drug Administration (FDA) approved biomaterial that can form nanosized micelles in aqueous solution [6]. TPGS micelles stand as an interesting system to perform drug delivery as they can carry lipophilic drugs and overcome P glycoprotein efflux as well [7, 8]. Therefore, TPGS combined with copolymers have been reported in many cancer research studies as a therapeutic drug carrier [6, 9–11]. The specific mechanism by which TPGS-based delivery systems reach tumoral tissue is not fully understood. However, many reports suggest there is an enhancement of therapeutic efficiency when TPGS is used either as TPGS-drug conjugates or TPGS with other copolymers. TPGS nanomicelles are most likely to accumulate rather specifically in tumors by vasculature fenestrations in angiogenic blood vessels or by specific targeting as folate, transferrin, or even antibody exposing TPGS-based drug delivery systems [12–14]. Most of the approaches are related to therapeutic interventions but its ability to reach tumoral tissue, possibly by EPR effect (passive targeting) or specific binding (active targeting), can also be exploited to develop imaging agents with diagnostic application.

Breast cancer is the most common cancer among women, accounting for the highest incidence and mortality rates worldwide [15]. Early and accurate diagnosis is needed to improve therapy efficacy and survival rates. Nuclear medicine imaging is useful in breast cancer at different instances: diagnosis confirmation, staging, and prediction of therapeutic response, which are all key factors in treatment decision. However, ^99m^Tc-sestamibi is the only routinely available single-photon emission computed tomography (SPECT) radiopharmaceutical used for mammary scintigraphy. In this regard, it is expected that the growing development in SPECT equipment and detection technology parallels with an increasing radioactive probes availability. In this way, the radiolabeling of TPGS micelles represents an interesting strategy to favor imaging probe development. We have previously reported a radiolabeling method for TPGS nanosized micelles and their biodistribution in a healthy animal model as well as their pharmacokinetics and stability *in vivo* [16]. The aim of this work was to evaluate the performance of this radioactive probe as a diagnostic imaging agent in two distinct breast cancer animal models compared to a routinely available SPECT radiopharmaceutical, ^99m^Tc-sestamibi.

## 2. Materials and Methods

### 2.1. Radiolabeling of Radioactive Probes

Radiolabeling of TPGS micelles (25 mg·mL^−1^) was performed by the direct method using SnCl_2_·H_2_O as reducing agent (7.5 *μ*g/mL) and 37–74 MBq (1–2 mCi) of ^99m^TcO_4_^−^ (obtained from a ^99^Mo-^99m^Tc column generator (BACON SAIC, Argentina), based on a previous work with slight modifications [16]. Saline solution (0.9%NaCl) was used as reaction solvent. Radiochemical purity (RCP) control was performed as follows: free ^99m^TcO_4_^−^ was measured by paper chromatography using acetone as mobile phase. Colloidal radioactive impurity was assessed by paper chromatography as well (using saline as mobile phase) and dynamic light scattering. For comparative purposes, ^99m^Tc-sestamibi was used. It was synthesized according to the manufacturer's instructions (BACON SAIC or Tecnonuclear SA, Argentina). Briefly, 2.2 GBq (60 mCi) of ^99m^TcO_4_^−^ was added to a commercial freeze-dried kit reaching a final volume of 3 mL with saline solution. Radiochemical purity was assessed as stated in United States Pharmacopoeia by reverse-phase thin layer chromatography and by solvent extraction method (chloroform) [17]. ^99m^Tc-sestamibi RCP was above 95% in all assays.

### 2.2. TPGS Micelles Size, Zeta Potential, and Morphological Characterization

^*99m*^ *Tc-radiolabeled TPGS micelles* were analyzed by transmission electronic microscopy (TEM) (Zeiss EM 109T equipped with a digital camera Gatan ES1000W, Germany). Samples were placed in a grid and covered with Formvar film. Finally, they were washed in distilled water and were dried in a silica gel support.

The average hydrodynamic diameter and size distribution of TPGS-radiolabeled micelles and colloidal radioactive impurity were measured by dynamic light scattering (DLS; Zetasizer Nano-ZSP, Malvern Instruments, UK) with a He-Ne laser (633 nm) and a digital correlator ZEN3600. Measurements were performed at a *θ* = 173° angle. Zeta potential was measured using the same analyzer, as described elsewhere [18]. Samples were previously equilibrated at 25°C, and results were expressed as the average of three measurements.

### 2.3. Cell Culture

4T1 Breast cancer murine cell line was obtained from American Type Culture Collection (ATCC® CRL-2539™ BALB/cfC3H strain). Cells were cultured in RPMI 1640 with fetal bovine serum 10% (FBS), glutamine 0.03%, fungizone 0.001%, and gentamicin 0.004% (Gibco BRL, EE.UU.) and were kept in an incubator at 37°C in a humidified atmosphere and 5% CO_2_.

### 2.4. ^9*9m*^*Tc-Sestamibi* and ^*99m*^*Tc-Radiolabeled TPGS Micelles* Uptake in 4T1 Breast Cancer Cell Line

To evaluate the cellular uptake of both radioactive probes, ^*99m*^*Tc-sestamibi* and ^*99m*^*Tc-radiolabeled TPGS micelles*, cells were grown over 6-well plastic plates until 70% of confluence was reached. Cells were then incubated with radioactive probes 0.37–0.74 MBq (10–20 *μ*Ci in 100 *μ*L) for 20, 40, 60, and 80 minutes in an incubator (5% CO_2_, at 37°C). Supernatant was removed and collected in plastic tubes. Cells were then washed with saline solution, and samples were kept for further measurements. Finally, cells were harvested using trypsin (0.25%w/v, 5 minutes). Radioactivity from supernatant, washed fractions and cellular fractions was measured in a well-type solid scintillating counter calibrated with corresponding standards (Alfanuclear, ZX, Argentina). Each time point was assayed in duplicate, and results of three independent experiments are shown as % total activity (added to the culture medium).

### 2.5. Experimental Breast Cancer Animal Models

Animal procedures were in accordance with international recommendations [19, 20], and protocols were approved by Ethical Committee for the Use and Care of Laboratory Animals of the School of Pharmacy and Biochemistry (Res D 3686/16).

BALB/c female mice (20–25 g) and female Sprague-Dawley rats (250–300 g) were purchased from the Division of Laboratory Animal Production, School of Pharmacy and Biochemistry, University of Buenos Aires (Argentina). Animals were kept in stainless steel cages with food and water ad libitum and 12 h light cycle.

#### 2.5.1. 4T1 Breast Cancer Model in Mice

The orthotopic 4T1 breast cancer model was established as described elsewhere [21]. Briefly, 4T1 cells were collected by centrifugation and resuspended in 100 *μ*L RPMI-1640. Tumors were induced by subcutaneous (s.c.) injection of the cells (1 × 10^5^) into the right upper mammary fat pad. Tumor diameters were measured with a caliper every other day until they reach 4 mm to perform imaging studies.

#### 2.5.2. Chemoinduced Breast Cancer Model in Rats

Mammary tumors were induced in Sprague-Dawley rats by the carcinogen *N*-nitroso-*N*-methyl urea (NMU) as described elsewhere [21]. Briefly, tumors were induced by three intraperitoneal (i.p.) injections (50 mg/Kg of body weight) of NMU (10 mg·mL^−1^ in saline solution) at 50, 80, and 110 days after rats' birth. The development of breast tumors was examined by palpation, three times a week, and they were detected from 90 days after the first administration of NMU. Tumor size was measured using a caliper every other day until they reach 8 mm to perform imaging studies.

### 2.6. Imaging Studies and Biological Distribution

Animals were anesthetized with isoflurane 2%v/v and O_2_ as carrier. *In vivo* uptake of ^99m^Tc-sestamibi or ^*99m*^*Tc-radiolabeled TPGS micelles* was evaluated by intravenous (i.v.) administration (tail vein) of 0.05–0.1 mL (3.7–37 MBq or 0.1–1 mCi/animal) to tumor-bearing animals in two sets of imaging studies performed with each radioactive probe in the same individual for rats (*N* = 3) and two different groups of mice (*N* = 8, 4 each group). After 15 min of biodistribution (^99m^Tc-sestamibi) or 12 hours (^*99m*^*Tc-radiolabeled TPGS micelles*), images were acquired with a small field of view gamma camera (OHIONUCLEAR, software: IM512P, ALFANUCLEAR, Argentina) equipped with a high-resolution parallel hole collimator which was used for ventral imaging. A 256 × 256 matrix was used with a 1.25 zoom for mice and 1.5 for rats. Acquisitions were performed for 15 minutes reaching at least 10^6^ counts. Images were visually analyzed in a band scale to optimize structure differentiation and identification.

An ex vivo study was performed to evaluate biodistribution of radioactive probes in BALB/c mice bearing 4T1 tumors. After imaging, mice were euthanized in a CO_2_ chamber, and organs of interest were excised, weighted, and measured in a well-type solid scintillation counter (Alfanuclear, ZX, Argentina) to express results as percentage of injected dose per gram of tissue (%ID/g).

In the case of NMU-induced tumor bearing rats, a semiquantitative analysis was performed by creating regions of interest (ROIs) over acquired images to evaluate radioactivity in the delimited area.

## 3. Results

### 3.1. TPGS Micelles Formation and Size Are Not Altered by ^99m^Tc Radiolabeling

**Figure 1:**
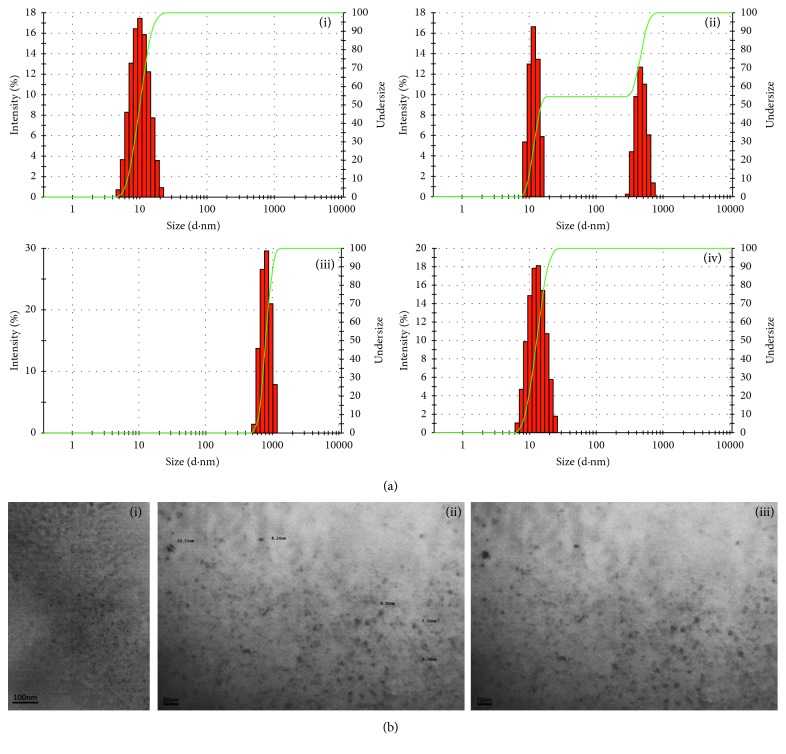
(a) Size distribution measured by dynamic light scattering (DLS). (i) TPGS nanomicelles; (ii) TPGS nanomicelles after ^99m^Tc radiolabeling; (iii) colloidal radioactive impurity; (iv) ^*99m*^*Tc-radiolabeled TPGS micelles* after 0.22 *μ*m filtration. (b) Transmission electronic microscopy photographs of ^*99m*^*Tc-radiolabeled TPGS micelles*. (i) Lower magnification; (ii) higher magnification and micelle size labels; (iii) higher magnification.

Radiolabeling of TPGS micelles by the direct method resulted in high yields since less than 5% of the activity was associated with free pertechnetate (^99m^TcO_4_^−^) in all experiments. Paper chromatography results, using saline as a mobile phase, revealed more than 98% of the activity at the origin. So, colloidal impurities, hydrolyzed and reduced ^99m^TcO_4_^−^, were not resolved by this technique. The use of SnCl_2_ as the reducing agent led to the formation of a ^99m^Tc colloidal impurity, extensively reported for ^99m^Tc radiolabeling protocols by the direct method [22]. DLS analysis allowed us to evaluate the presence of that impurity. A second population was found in the reaction product sample assessed by DLS (Figure 1(a) (ii) compared to Figure 1(a) (i)). Population identity was confirmed by performing an alternative radiolabeling protocol, in absence of ligand (colloidal formation is highly favored in this reaction conditions). This radiolabeling product was also analyzed by DLS as it is shown in Figure 1(a) (iii): a colloidal/particulate structure was found, and it could be assigned to radioactive impurity by its size (600 nm) as it matches the second population size found in ^*99m*^*Tc-radiolabeled TPGS micelles* sample. Since colloidal impurity showed a hydrodynamic diameter significantly higher than ^*99m*^*Tc-radiolabeled TPGS micelles*, separation by filtration (0.22 *μ*m methyl cellulose esters, Merck, Germany) was possible. Filtrated and retained radioactivity was measured for both radiolabeling protocols: (i) TPGS radiolabeling with ^99m^Tc and (ii) ^99m^Tc radioactive colloid favoring radiolabeling conditions. Filtration results are shown in Table 1. Almost half the radioactivity was associated with colloidal impurity, which was retained in the filter. Moreover, TPGS nanosized micelles were conserved and retained their radiolabel after filtration, as it can be concluded by results shown in Figure 1(a) (iv) and Table 1. Finally, ^*99m*^*Tc-radiolabeled TPGS micelles* were characterized regarding size and morphology by DLS and TEM imaging showing spherical structures with an average diameter of 9.58 nm and polydispersion index of 0.09 at 25°C (Figure 1(a) (iv) and Figure 1(b)). Zeta potential resulted in a −0.217 ± 1.54 mV at 25°C, as well.

### 3.2. Breast Cancer Animal Model of 4T1 Cell Line Yielded Negative Imaging Results for ^*99m*^*Tc-Radiolabeled TPGS Micelles* and ^99m^Tc-Sestamibi

**Figure 2:**
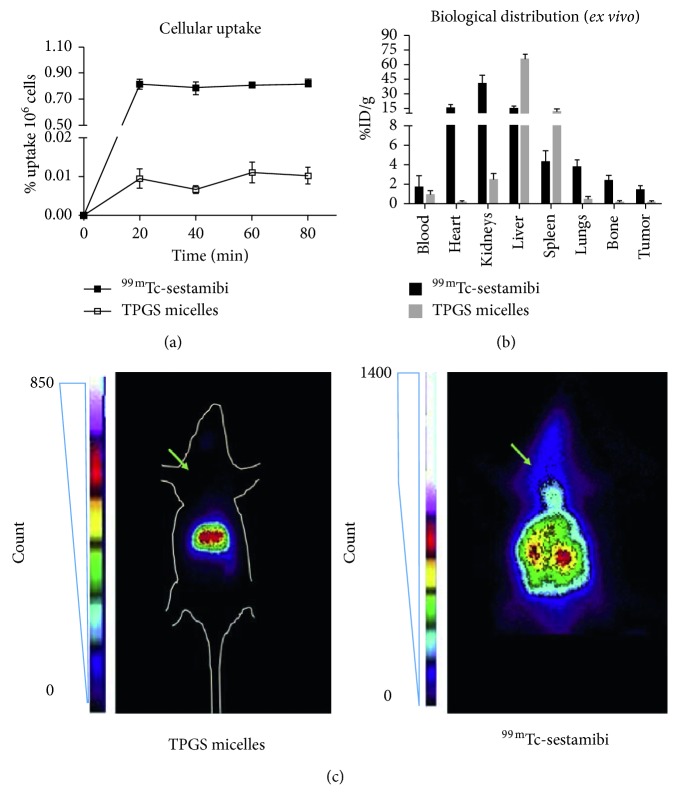
(a) ^99m^Tc-sestamibi and ^*99m*^*Tc-radiolabeled TPGS micelles* cellular uptake (4T1 cell line). Results are expressed as the percentage of activity accumulated in the cellular fraction normalized by 10^6^ cells. (b) ^99m^Tc-sestamibi and ^*99m*^*Tc-radiolabeled TPGS micelles* biodistribution in a breast cancer animal model developed with 4T1 murine cell line in BALB/c mice. Results are expressed as the percentage of injected dose in each organ normalized by organ weight (%ID/g). Bars represent mean ± SD. (c) Gamma camera imaging of ^*99m*^*Tc-radiolabeled TPGS micelles* (12 h after injection) and ^99m^Tc-sestamibi (15 min after injection) in a breast cancer animal model developed with 4T1 murine cell line in BALB/c mice. Green arrows indicate the palpable tumoral site. Representative images are shown. Mice outline was drawn for anatomical references.

Cellular uptake of ^*99m*^*Tc-radiolabeled TPGS micelles* was assessed in 4T1 murine breast cancer cell line. Accumulation of this radioactive probe in the cellular fraction was not significant. However, routinely available and commercial ^99m^Tc-sestamibi was also evaluated and resulted in less than 1% of the activity accumulated in 4T1 cells. *In vitro* results can be seen in Figure 2(a).

*In vivo* results revealed the inability of ^*99m*^*Tc-radiolabeled TPGS micelles* to allow tumor localization by gamma camera imaging in 4T1 breast cancer animal model. Images showed a strong hepatic uptake, but no signal was detected in tumor palpable site (shown by green arrows in Figure 2(c)) or any other organ. Comparatively, no tumoral site was evidenced by gamma camera imaging using ^99m^Tc-sestamibi either. *Ex vivo* biodistribution results confirmed the elevated percentages of ^*99m*^*Tc-radiolabeled TPGS micelles* in the liver and spleen predominantly (Figure 2(b)). Kidneys uptake is also evident *ex vivo*, although they are not markedly distinguished in gamma camera imaging, probably due to superposition in planar acquisition.

^99m^Tc-sestamibi biodistribution was in accordance with previous reports and extensive radiopharmaceutical use and knowledge [23, 24]. Heart, kidneys, lungs, bone, and tumor uptake was higher for ^99m^Tc-sestamibi than for ^*99m*^*Tc-radiolabeled TPGS micelles*. However, specifically for tumoral site, the difference in radioactive probe accumulation was not enough to be evidenced by acquired images. *Ex vivo* results are shown in Figure 2(b).

### 3.3. ^*99m*^*Tc-Radiolabeled TPGS Micelles* Outperform ^99m^Tc-Sestamibi in Tumoral Visualization by Gamma Camera Imaging in Breast Cancer Animal Model Induced by NMU

**Figure 3:**
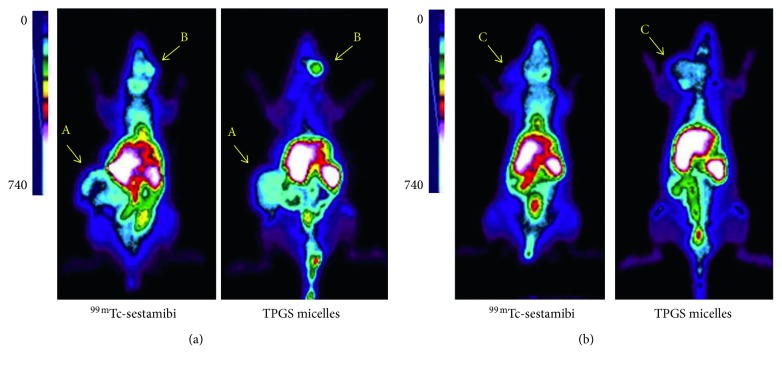
Gamma camera imaging of ^99m^Tc-sestamibi (15 min after injection) and ^*99m*^*Tc-radiolabeled TPGS micelles* (12 h after injection) in a breast cancer animal model developed with NMU carcinogen in Sprague-Dawley rats. Each animal was scanned with both radioactive probes, and representative images from (a) rat#1 and (b) rat#2 are shown. Yellow arrow indicates palpable tumoral site; A, B, and C represent tumor identification on each rat.

Acquired images are shown in Figure 3. ^99m^Tc-sestamibi biodistribution was consistent with previous results from our laboratory [24] and others [25, 26]. All palpable tumors were visualized by gamma camera imaging. Qualitative analysis revealed a higher tumoral site accumulation for ^*99m*^*Tc-radiolabeled TPGS micelles* compared to ^99m^Tc-sestamibi, resulting in a better imaging signal (increased counts). Additionally, a semiquantitative analysis was assayed by drawing regions of interest in tumor and background areas to calculate tumor/background ratio shown in Table 2. This ratio was at least 3 times higher for ^*99m*^*Tc-radiolabeled TPGS micelles* compared with ^99m^Tc-sestamibi. Interestingly, necrotic areas in tumor core noted by ^99m^Tc-sestamibi (Figure 3, tumor A) scintigraphy showed homogeneous ^*99m*^*Tc-radiolabeled TPGS micelles* uptake.

## 4. Discussion

The radiolabeling of nanosystems, especially with ^99m^Tc, presents some challenges as the chemistry behind this radionuclide incorporation is not fully understood. More importantly, radiochemical impurities can appear as a consequence of the radiolabeling process. The colloidal nature of those impurities represents an interference that jeopardizes result interpretation. Nevertheless, particulate commercial radiopharmaceuticals do not have a specific radiochemical assay to evaluate the presence of ^99m^Tc colloid. Additionally, there is limited information and reports regarding this issue [27, 28]. In this work we provided, not only a method to evaluate colloidal impurities but also valuable information in order to eliminate it from the reaction product. Moreover, purification by a filtration method using a 0.22 *μ*m filter is compatible with routine nuclear pharmacy procedures during radioactive probe synthesis. Size, zeta potential, and morphology of radiolabeled-TPGS nanomicelles were also assessed, and though these results may not explain entirely *in vivo* results, they are part of the radioactive probe's characterization.

Then, pure ^*99m*^*Tc-radiolabeled TPGS micelles* were evaluated for their performance as breast cancer diagnostic agent compared with a commercially available SPECT radiopharmaceutical, which is routinely used. ^99m^Tc-sestamibi was firstly introduced in clinical area more than 25 years ago in the United States (FDA approved in 1990) as a radioactive probe useful in myocardial perfusion assessment. Simultaneously, during the 90s, many publications reported a preferential uptake of this radiopharmaceutical by cancer cell lines with respect to normal ones [29–31]. In 2001, a commercial formulation, with specific use and indication for breast cancer diagnosis, became available, although it is currently discontinued. Since then, ^99m^Tc-sestamibi has been investigated as a diagnostic probe in several types of cancer, for mammary scintigraphy and parathyroid adenoma imaging [32–36]. The development of new techniques such as breast specific gamma imaging (BSGI) has revalued the usefulness of ^99m^Tc-sestamibi in oncology field [37–40]. Furthermore, the ability of this radiopharmaceutical to predict or correlate with molecular subtypes of breast cancer has been very recently explored [41]. The evidence described above indicates that ^99m^Tc-sestamibi is a suitable SPECT radiopharmaceutical to be used for comparative purposes in gamma imaging.

*In vitro* results revealed that there is no ^*99m*^*Tc-radiolabeled TPGS micelles* uptake in 4T1 breast cancer cell line. In the same way, 4T1 animal model in BALB/c mice showed negative gamma camera images. That is, palpable tumors were not seen in the acquired images. Therefore, it can be concluded that this radioactive probe does not reach tumoral site neither at a cellular level nor at tumoral interstice. Biodistribution of ^*99m*^*Tc-radiolabeled TPGS micelles* can be explained by the very small size of the micelles, which would be responsible for phagocytic hepatic uptake by reticuloendothelial system and could also be a consequence of interactions with blood elements, such as opsonization, which could lead to liver accumulation [42, 43].

Since *in vivo* results have many variables affecting tumoral uptake such as perfusion, lymphatic drainage, and blood pool recirculation; among others, there is no way to identify which ends up determining the net accumulation. Nevertheless, the absence of gamma camera detection, that is, a negative image, says enough about ^99m^Tc nanosized micelles performance as a diagnosis agent in 4T1 breast cancer animal model. Noticeably, 4T1 *in vitro* cellular uptake was higher for ^99m^Tc-sestamibi but it was still a very poor accumulation (lower than other breast cancer cell lines, such as human MDA-MB-231 and MCF-7, data not shown). In this line, commercially available and routinely used ^99m^Tc-sestamibi rendered negative images in the breast cancer animal model of 4T1.

On the other hand, very different results were found in gamma camera imaging of NMU-induced tumor bearing animals. All tumors from NMU breast cancer animal model were found to be epithelial neoplasias, particularly invasive carcinomas. However, each had different histologic type (data not shown) [44]. Therefore, for comparative purposes, we imaged each animal with both radioactive probes. Noninvasive gamma imaging proved to be useful in this sense. From a diagnostic perspective, the usefulness and information provided by imaging preclinical studies are of utmost importance in imaging probe development. In this case, both radioactive probes were able to show tumor localization. However, diagnostic performance was significantly different. ^*99m*^*Tc-radiolabeled TPGS micelles* had an enhanced tumor uptake since every tumor was visualized by gamma camera imaging. Additionally, the tumor/background ratio in each tumor resulted at least 3 times higher for ^*99m*^*Tc-radiolabeled TPGS micelles* than ^99m^Tc-sestamibi. Interestingly, one tumor showed a cold core in ^99m^Tc-sestamibi image, which was further assigned to a necrotic area (data not shown) but a homogeneous uptake was observed for ^*99m*^*Tc-radiolabeled TPGS micelles* image. These results are the baseline to conclude that, with reference to NMU chemically induced breast cancer animal model, ^*99m*^*Tc-radiolabeled TPGS micelles* are superior than ^99m^Tc-sestamibi as a diagnostic agent for gamma camera imaging.

Further studies are needed to validate ^*99m*^*Tc-radiolabeled TPGS micelles* tumoral uptake in other breast cancer animal models, test their sensitivity and specificity as a breast cancer diagnostic agent, and determine the dosimetry associated with its biodistribution.

## 5. Conclusions

We conclude that ^*99m*^*Tc-radiolabeled TPGS micelles* might be a useful diagnostic agent for gamma camera imaging, presenting proper diagnostic characteristics in NMU-induced breast cancer animal, even detectable in necrotic areas. It is important to highlight that the presented radiolabeling method consists in a procedure that is perfectly compatible with nuclear medicine service practices.

## Figures and Tables

**Table 1:** Activity presence in filtered radiolabeling products (0.22 *μ*m).

	TPGS radiolabeling with ^99m^Tc	Radiolabeling with ^99m^Tc in colloidal radioactive impurity favoring conditions
% filtered activity	44 ± 4	0.7 ± 0.2

Results are expressed as the percentage of the initial activity that was measured in the filtrated fraction (filtered activity/initial activity *∗* 100). The mean ± SD of three independent radiolabeling reactions is shown.

**Table 2:** Semiquantitative analysis of gamma camera imaging of tumors from NMU breast cancer animal model.

Tumor identification	Tumor/background ratio		
	^*99m*^ *Tc-radiolabeled TPGS micelles*	^99m^Tc-sestamibi	Increase factor
A	79.6	26.3	3
B	11.5	2.6	4
C	22.3	3.9	6

## Data Availability

The data used to support the findings of this study are included within the article.
